# High Photosynthetic Photon Flux Density Differentially Improves Edible Biomass Space Use Efficacy in Edamame and Dwarf Tomato

**DOI:** 10.3390/plants13131858

**Published:** 2024-07-05

**Authors:** Qingxin Liu, Xinglin Ke, Eiji Goto

**Affiliations:** 1 Graduate School of Horticulture, Chiba University, Matsudo 648, Matsudo 271-8510, Chiba, Japan; 2 Research Center for Space Agriculture and Horticulture, Chiba University, Matsudo 648, Matsudo 271-8510, Chiba, Japan

**Keywords:** accumulation volume, dry weight fraction, plant factory with artificial light, yield, vertical farm

## Abstract

Improving edible biomass space use efficacy (EBSUE) is important for sustainably producing edamame and dwarf tomatoes in plant factories with artificial light. Photosynthetic photon flux density (PPFD) may increase EBSUE and space use efficacy (SUE). However, no study has quantitatively explained how PPFD affects EBSUE in edamame and dwarf tomatoes. This study aimed to quantitatively validate the effects of PPFD on EBSUE in dwarf tomatoes and edamame and verify whether this effect differs between these crops. The edamame and dwarf tomato cultivars ‘Enrei’ and ‘Micro-Tom’, respectively, were cultivated under treatments with PPFDs of 300, 500, and 700 µmol m^−2^ s^−1^. The results showed that the EBSUE and SUE increased with increasing PPFD in both crops. The EBSUE increased depending on the increase in SUE, the dry mass ratio of the edible part to the total plant in the edamame, and the SUE only in the dwarf tomatoes. In conclusion, a high PPFD can improve the EBSUE and SUE of edamame and dwarf tomatoes in different ways at the reproductive growth stage. The findings from this study offer valuable information on optimizing space and resource usage in plant factories with artificial light and vertical farms. Additionally, they shed light on the quantitative impact of PPFD on both EBSUE and SUE.

## 1. Introduction

Plant factories with artificial light (PFALs) and vertical farms with multi-layered cultivation systems illuminated artificially have been extensively utilized to consistently produce fresh, high-quality agricultural products year-round [1,2,3]. PFALs can control environmental factors and parameters such as light, air temperature and humidity, nutrient solution temperature, and CO_2_ concentration to enhance plant growth. Furthermore, compared to greenhouses and fields, PFALs save both water and CO_2_ during production [4].

In a PFAL, a significant production cost is the electric energy consumed by lamps and its air conditioning system for dehumidifying, cooling, and heating [4]. Energy consumption can be reduced by using higher-electric-efficiency lamps and higher-performance air conditioning systems. Reducing the cultivation shelf space in multilayered cultivation systems in PFALs is also a good means of reducing the running costs of air conditioning because the volume of the targeted air is controlled, and the cooling and heating load of the air conditioning system can be lowered. Space use efficacy (SUE) is described as the total dry biomass produced per unit of the accumulated cultivation volume during a given growth period, combining both the dry biomass production and cumulative volume occupied by crops. Edible biomass space use efficacy (EBSUE), defined as the crop edible biomass produced per unit of cumulative volume occupied by the crops during a growth period, is a more meaningful indicator. According to these definitions, SUE and EBSUE are can be improved by reducing cultivation space volume and/or increasing crop dry biomass. The volume of space needed for producing crops is influenced by the shape of the crop produced, with plant height being the most significant factor. To utilize space more efficiently, shorter plants allow for increasing the number of layers of plants in a multilayered cultivation system.

In the present study, vegetable soybean (edamame; *Glycine max* (L.) Merrill) and dwarf tomato (*Lycopersicon esculentum*) were selected, and their EBSUEs were compared for different photosynthetic photon flux densities (PPFDs). Edamame contains a higher vitamin content and fewer indigestible oligosaccharides compared to grain-type soybeans [5,6]. In addition, the cultivation period of edamame is shorter than that of grain-type soybeans. Therefore, fresh and agrichemical-free edamame can be a candidate crop for a commercial PFAL in the near future. The present study examined edamame, which is representative of high-stem crops (such as wheat and cucumber). Tomatoes contain high vitamin C levels and lycopene, and dwarf tomatoes also have potential advantages in high-efficiency PFAL cultivation [7]. Therefore, dwarf tomatoes, which are representative of short-stem crops (such as sweet potato and strawberry), were also investigated in the present study. To date, no research has investigated improving EBSUE in edamame and dwarf tomatoes in PFALs.

Light is an important environmental factor for plant growth and development. As an essential light intensity parameter, PPFD (with wavelengths between 400 and 700 nm) has the potential to influence SUE through its impact on variables such as biomass production [8,9], plant height [10,11], and projected leaf area [12,13]. Earlier findings suggest that shading conditions or a decreased PPFD significantly decrease the numbers of pods and seeds, the seed weight, and the seed yield of soybean [14,15]. These previous studies on plant responses to shading have investigated the allocation pattern of the matter in the whole plant and its morphological plasticity.

A previous study showed that PPFD can affect plant height, petiole length, and leaf angle [16]. Ke et al. [9] demonstrated that increasing PPFD (from 200 to 700 μmol m^−2^ s^−1^) increased the fresh and dry weights of dwarf tomatoes (Micro-Tom) and decreased their specific leaf areas. They also found that the dry mass allocated to fruits decreased with increasing PPFD. Yan et al. [17] reported that the dry matter partitioning and fruit yields of tomatoes (cultivar: ‘Ruifen882’) increased with a supplementary light treatment (daily light integral (DLI) ~16.78 mol m^−2^ d^−1^) compared with no supplementary treatment (DLI ~12.38 mol m^−2^ d^−1^). Therefore, PPFD may be a suitable environmental factor for regulating EBSUE by influencing dry biomass distribution and plant height. However, no prior studies have systematically and quantitatively explained how PPFD affects EBSUE in edamame and dwarf tomatoes. 

This study examines the effects of PPFD on the EBSUEs in two non-leaf vegetable crops, edamame and dwarf tomatoes, aiming to establish a foundation for achieving high-efficiency cultivation in PFALs. We hypothesized that the PPFD would have different effects on the EBSUE in edamame and tomatoes by affecting their dry mass distribution and accumulated volume differently. This study had two specific purposes. The first was to quantitatively study the effects of PPFD on EBSUEs in dwarf tomatoes and edamame. The second was to elucidate the mechanism causing these phenomena between the two crops by considering both morphological and physiological aspects.

## 2. Results

### 2.1. EBSUE and SUE

**Figure 1:**
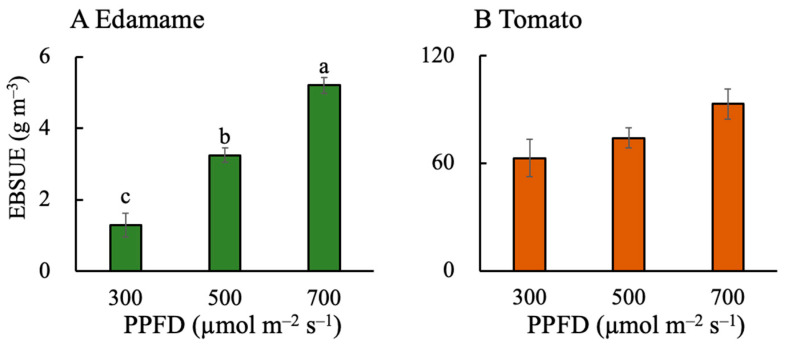
Effects of photosynthetic photon flux density (PPFD) on edible biomass space use efficacy (EBSUE) in edamame (**A**) and dwarf tomatoes (**B**). Error bars indicate standard error (*n* = 4). Different letters above the error bars indicate significant differences, determined using Tukey’s HSD test at *p* < 0.05.

**Figure 2:**
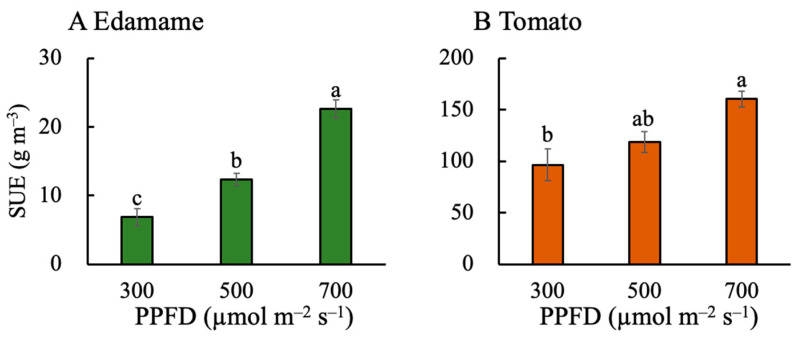
Effects of photosynthetic photon flux density (PPFD) on space use efficacy (SUE) in edamame (**A**) and dwarf tomatoes (**B**). Error bars indicate standard error (*n* = 4). Different letters above the error bars indicate significant differences, determined using Tukey’s HSD test at *p* < 0.05.

The EBSUE value increased with increasing PPFD in both the edamame and dwarf tomatoes (Figure 1). The EBSUE values were 1.95 and 3.91 g m^−3^ higher at PPFDs of 500 and 700 μmol m^−2^ s^−1^ (E500 and E700), respectively, than that at a PPFD of 300 μmol m^−2^ s^−1^ (E300) (Figure 1A). In addition, the EBSUE values were 11.17 and 30.22 g m^−3^ higher at PPFDs of 500 and 700 μmol m^−2^ s^−1^ (T500 and T700), respectively, than that at a PPFD of 300 μmol m^−2^ s^−1^ (T300) (Figure 1B). The SUE values also increased with increasing PPFD in both the edamame and dwarf tomatoes (Figure 2). The SUE values were 5.48 and 15.79 g m^−3^ higher for E500 and E700, respectively, than that for E300 (Figure 2A). Additionally, the SUE values were 22.36 and 64.15 g m^−3^ higher for T500 and T700, respectively, than that for T300 (Figure 2B). The EBSUE and SUE values were higher in the dwarf tomatoes than those in the edamame at the same PPFD (Figure 1 and Figure 2).

### 2.2. Edible and Above-Ground Dry Weight and Dry Weight Fraction

**Figure 3:**
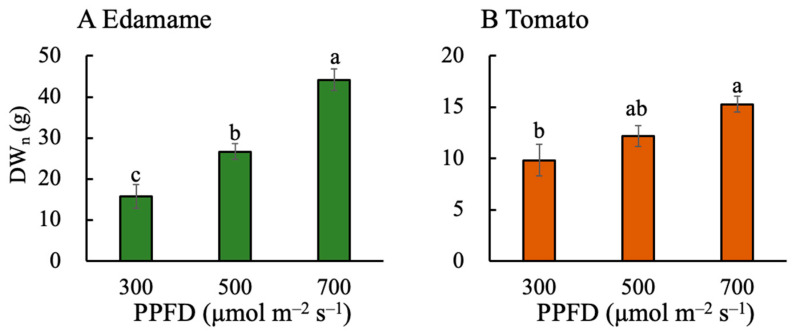
Effects of photosynthetic photon flux density (PPFD) on above-ground dry weight (DW_n_) in edamame (**A**) 66 d after sowing (DAS) and tomato (**B**) 82 DAS. Error bars indicate standard error (*n* = 4). Different letters above the error bars indicate significant differences, determined using Tukey’s HSD test at *p* < 0.05.

**Figure 4:**
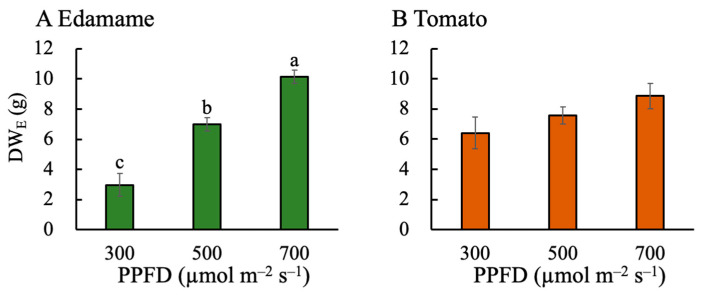
Effects of photosynthetic photon flux density (PPFD) on edible dry weight (DW_E_) in edamame (**A**) 66 d after sowing (DAS) and dwarf tomatoes (**B**) 82 DAS. Error bars indicate standard error (*n* = 4). Different letters above the error bars indicate significant differences, determined using Tukey’s HSD test at *p* < 0.05.

The above-ground dry weight (DW_n_) increased with increasing PPFD in the edamame and dwarf tomatoes (Figure 3). The DW_n_ was higher in the edamame than in the dwarf tomatoes at the same PPFD. The DW_n_ values were 10.87 and 28.37 g higher for E500 and E700, respectively, than that for E300 (Figure 3A). Furthermore, the DW_n_ values were 2.33 and 5.45 g higher for T500 and T700, respectively, than that for T300 (Figure 3B). The edible dry weight (DW_E_) also increased with increasing PPFD (Figure 4). At PPFDs of 500 and 700 μmol m^−2^ s^−1^, the DW_E_ values of the dwarf tomatoes were higher than those of the edamame. However, the DW_E_ value of the edamame was higher than that of the dwarf tomatoes at a PPFD of 700 μmol m^−2^ s^−1^. The DW_E_ values were 4.03 and 7.18 g higher for E500 and E700, respectively, than that for E300 (Figure 4A). Moreover, the DW_E_ values were 10.87 and 28.37 g higher for T500 and T700, respectively, than that for T300 (Figure 4A).

**Figure 5:**
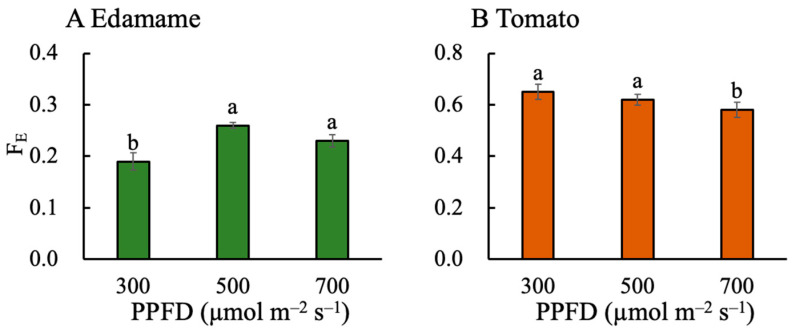
Effects of photosynthetic photon flux density (PPFD) on the fraction of dry mass partitioned to edible organs (F_E_) in edamame (**A**) 66 d after sowing (DAS) and dwarf tomatoes (**B**) 82 DAS. Error bars indicate standard error (*n* = 4). Different letters above the error bars indicate significant differences, determined using Tukey’s HSD test at *p* < 0.05.

The fraction of dry mass partitioned to edible organs (F_E_) first increased and then decreased in the edamame as the PPFD increased (Figure 5A). The F_E_ values were 0.07 and 0.04 g g^−1^ higher for E500 and E700, respectively, than that for E300. Higher PPFDs led to lower F_E_ values in the dwarf tomatoes (Figure 5B). The F_E_ values were 0.03 and 0.06 g g^−1^ lower for T500 and T700, respectively, than that for T300. The F_E_ of the dwarf tomatoes was higher than that of the edamame at the same PPFD (Figure 5). 

**Figure 6:**
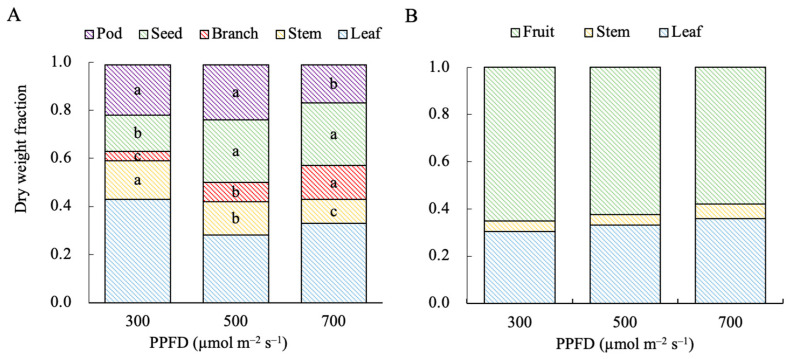
Effects of photosynthetic photon flux density (PPFD) on the dry weight fraction of each organ in edamame (**A**) 66 d after sowing (DAS) and dwarf tomatoes (**B**) 82 DAS. Different letters indicate significant differences between treatments according to Tukey’s HSD test conducted independently for pods, seeds, branches, stems, and leaves (*n* = 4, *p* < 0.05).

In the edamame plants, higher PPFDs led to higher and lower dry weight fractions of their branches and stems, respectively (Figure 6A). The fraction of dry mass partitioned to leaves first decreased and then increased as the PPFD increased. In the dwarf tomatoes, higher PPFDs led to higher stem and leaf dry weight fractions, but these were lower than those of fruits (Figure 6B).

### 2.3. Accumulated Cultivation Volume and Plant Height

**Figure 7:**
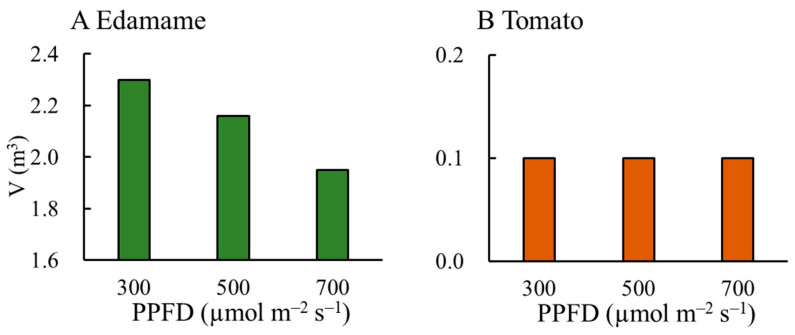
Effects of photosynthetic photon flux density (PPFD) on accumulated cultivation volume (V) in edamame (**A**) and dwarf tomatoes (**B**).

**Figure 8:**
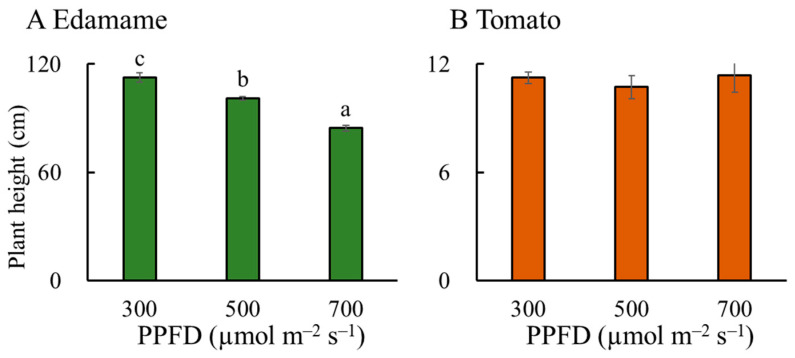
Effects of photosynthetic photon flux density (PPFD) on plant height in edamame (**A**) 66 d after sowing (DAS) and dwarf tomatoes (**B**) 82 DAS. Error bars indicate standard error (*n* = 4). Different letters above the error bars indicate significant differences, determined using Tukey’s HSD test at *p* < 0.05.

The accumulated cultivation volume (V) decreased with increasing PPFD in the edamame (Figure 7A). The V values were 0.14 and 0.35 m^3^ lower for E500 and E700, respectively, than that for E300. However, the PPFD hardly affected the V values in the dwarf tomatoes (Figure 7B). The V values in the edamame were much greater than those in the dwarf tomatoes (Figure 7). Similar to the V, the plant height also decreased with increasing PPFD in the edamame 66 d after sowing (DAS) (Figure 8A) but had hardly changed in the dwarf tomatoes 82 DAS (Figure 8B).

### 2.4. Photosynthetic Capacity

**Figure 9:**
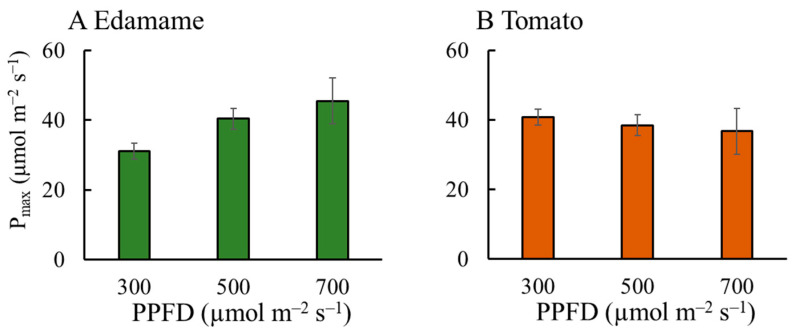
Effects of photosynthetic photon flux density (PPFD) on photosynthetic capacity (maximum net photosynthetic rate (P_max_)) in edamame (**A**) 36 d after sowing (DAS) and dwarf tomatoes (**B**) 64 DAS. Error bars indicate standard error (*n* = 4).

The maximum net photosynthetic rate (P_max_) increased with increasing PPFD in the edamame (Figure 9A). The P_max_ values increased by 30 and 46% for E500 and E700, respectively, relative to that for E300. The P_max_ decreased with increasing PPFD in the dwarf tomatoes (Figure 9B). The P_max_ values decreased by 6 and 11% for T500 and T700, respectively, relative to that for T300. At a PPFD of 300 μmol m^−2^ s^−1^, the P_max_ value in the dwarf tomatoes was higher than that in the edamame (Figure 9). However, at PPFDs of 500 and 700 μmol m^−2^ s^−1^, the P_max_ values of the dwarf tomatoes were lower than those of the edamame.

## 3. Discussion

### 3.1. High PPFD Leads to High EBSUE by Increasing Both SUE and F_E_ in Edamame

The high PPFDs (500 and 700 μmol m^−2^ s^−1^) improved the EBSUE (Figure 1A) in the edamame by increasing the SUE (Figure 2A) and F_E_ (Figure 5A). In fact, its SUE rather than F_E_ was the main reason for its improved EBSUE (Figure S1). Furthermore, increasing the PPFD improved the SUE by improving the DW_n_ (Figure 3A) and decreasing the V (Figure 7A). The effect of PPFD on the DW_n_ was higher than that on the V (Figure S2). 

For the cultivation space, the reduction in V at a higher PPFD was mainly due to the reduction in plant height (Figure 8A). In addition, a greater amount of dry mass was distributed in branches and seeds rather than stems with increasing PPFD (Figure 6A), which made the stems thinner. Hitz et al. [16] found that a low PPFD (100 μmol m^−2^ s^−1^) increased stem length by affecting the internode elongation of soybeans. Our results showed that the number of nodes did not increase in the E300 treatment compared to that in the E700 treatment (Table S1). Other studies have also confirmed that low-PPFD conditions promote the upward growth of stems and petioles for cultivars of *Helianthus annuus* and *Arabidopsis thaliana* [18,19,20]. Feng et al. [15] found that soybeans’ stem length decreased and their stem diameter increased when the PPFD increased from 100 to 500 μmol m^−2^ s^−1^. It has been argued that a low PPFD alters the photosynthetic system as well as the stem carbohydrate concentration and composition [21,22]. ‘Enrei’, a soybean cultivar, has a high protein content and wide-area adaptability and can be cultivated at a high density [23]. In the present study, the plant height of ‘Enrei’ was affected by the PPFD, but other edamame varieties, especially dwarf varieties, may not be affected by the PPFD; this requires further research.

Regarding dry biomass accumulation, a high PPFD promoted photosynthesis and, thereby, dry matter accumulation. Additionally, the P_max_ increased with increasing PPFD in the edamame (Figure 9A). P_max_ is an important parameter for describing photosynthetic ability and determining the shape of the light response curve of leaves [24,25,26]. Bowes et al. [27] argued that higher light intensities in soybean lead to higher photosynthetic rates. In addition, a higher PPFD led to a lower intercellular CO_2_ concentration, which also improved Pn and P_max_ (Figure S3).

Seeds and pods are important factors determining soybean yield [28], and a high PPFD has been found to increase the numbers of seeds and pods [29,30], which is consistent with our results (Table S1 and Figure 4A). 

### 3.2. Effects of PPFD on F_E_, V, Plant Height, and P_max_ in Dwarf Tomatoes

In the dwarf tomatoes, the higher PPFD resulted in a decreased F_E_ (Figure 5B). However, a higher PPFD increased their SUE (Figure 2B), counteracting this negative effect and increasing EBSUE overall (Figure 1B). Contrastingly, one study found that the F_E_ in indeterminate tomatoes increased with increasing PPFD [17], while another study of determinate ‘Micro-Tom’ tomatoes found that the number of fruits on the main stem and fruit sink strength limited their F_E_ [9]. This limitation in fruit number explains why the F_E_ did not increase with the PPFD in the present study. The PPFD positively affected the DW_n_ (Figure 3B) but had a negligible effect on the V (Figure 7B) in the tomatoes. Ultimately, the positive effect on the DW_n_ led to a positive effect of the PPFD on their SUE. Similar to the findings in the edamame, the DW_n_ was higher at a higher PPFD because of a higher photosynthetic rate, consistent with previous studies [31,32]. 

Unlike the findings in the edamame, a high PPFD hardly influenced tomato plant height (Figure 8B) and thus did not affect the V. However, Wei et al. [33] argued that the stem length of tomatoes (*S. lycopersicum* L. ‘Super Sunload’ and ‘Super Dotaerang’) increased with increasing PPFDs from 50 to 150 μmol m^−2^ s^−1^. Moreover, Zheng et al. [32] found that tomato (*S. lycopersicum* L. ‘Hakumaru’) plant height increased with increasing PPFDs from 60 to 240 μmol m^−2^ s^−1^ and decreased with further increases in PPFDs from 240 to 330 μmol m^−2^ s^−1^. Indeed, at the reproductive growth stage, PPFD hardly affects the plant height of dwarf cultivars with recessive mutations in *sp* (*self-pruning*), *d* (*dwarf*), and *mnt* (*miniature*) genes [34,35,36]. Collectively, these studies showed that the effect of PPFD on plant height varies depending on the tomato variety and growth stage. 

### 3.3. Effects of PPFD on F_E_, V, Plant Height, and P_max_ in Edamame and Dwarf Tomatoes

The PPFD similarly affected the EBSUE, SUE, DW_n_, and DW_E_ in the edamame and dwarf tomatoes. An increased PPFD resulted in higher EBSUEs in both the edamame and dwarf tomatoes primarily because of the increasing SUE. However, at high PPFDs, the F_E_ in the edamame improved, but it decreased in the dwarf tomatoes because the number of edible organs in the dwarf tomatoes was limited, unlike in the edamame. These results verified our hypothesis. The effect of PPFD on DW_n_ was larger than that on the V in both the edamame and dwarf tomatoes (Figure S2). The PPFD has different effects on the V because of plant height. When the PPFD increased from 300 to 700 μmol m^−2^ s^−1^, the tomato and soybean leaves exhibited a converse P_max_ response (Figure 9). Cai and Xu [37] reported that at a PPFD of 700 μmol m^−2^ s^−1^ for a duration of 3 h, the proportion of PSII dimer to total PSIIs, D1 protein level, and light saturation rate of PSII electron transport in soybean leaves did not change significantly, indicating that, for soybean leaves at a PPFD of 700 μmol m^−2^ s^−1^, the reversible downregulation had a sufficient capacity to protect PSII from photodamage. However, for tomato leaves exposed to 700 μmol m^−2^ s^−1^, the PSII electron transport rate decreased, indicating that the plant had an insufficient capacity to protect PSII from photodamage [9]. Regardless, the PPFD at 700 μmol m^−2^ s^−1^ did not inhibit dry biomass accumulation in the tomatoes (Figure 3B); however, the P_max_ decreased at a higher PPFD (Figure 9B). In addition, a high PPFD caused more dry mass partitioning to leaves and stems in the tomatoes than in the edamame (Figure 6 and Tables S1 and S2). 

Although the effects of PPFD on the SUE, F_E_, DW_n_, V, EBSUE, and plant height (Figures S1, S2 and S4) in the edamame were larger than those in the dwarf tomatoes, the EBSUE and SUE in the dwarf tomatoes were much higher than those in the edamame (Figure 1 and Figure 2) owing to their height advantage over normal edamame cultivars, resulting in decreased crop cultivation space requirements. Therefore, dwarf cultivars have more space utilization advantages than general tomato cultivars. In the future, dwarfing genes may contribute greatly to breeding engineering for high-efficacy cultivation in PFALs.

In the present study, we only selected one cultivar each for edamame (‘Enrei’) and dwarf tomato (‘Micro-Tom’). However, the effects of PPFD on EBSUE and SUE may differ depending on cultivars. Additionally, future studies should examine the effects of PPFD on EBSUE and SUE in candidate crops cultivated in PFALs. The effects of PPFD on the SUE or EBSUE in long-stem crops such as cucumber may be similar to those in ‘Enrei’; similarly, those in short-stem crops (such as strawberry (*Fragaria* × *ananassa*) or dwarf cultivars) may be similar to those in ‘Micro-Tom’. Therefore, the effects of PPFD on EBSUE in other cultivars and crops should be studied in the future. Moreover, in determining the EBSUE and SUE, the SUE of underground plant parts was not considered. Since nutrients and water are mainly absorbed through the roots, underground plant parts may affect dry matter accumulation, SUE, and EBSUE and therefore should be studied in the future.

## 4. Materials and Methods

### 4.1. Plant Materials and Growth Conditions

**Figure 10:**
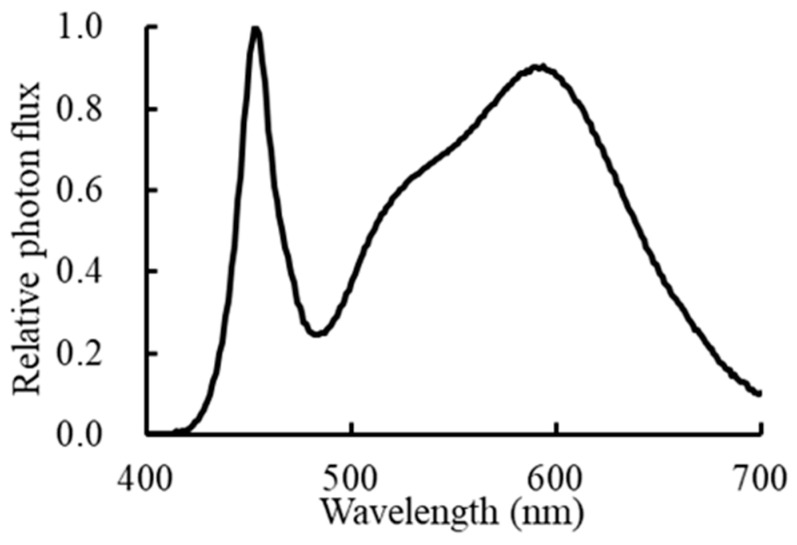
Photon flux distribution of white LED lamps (XLX450NHNU LE9; Panasonic Corporation, Osaka, Japan). The maximum photon flux value was converted to 1.0. Spectra were determined using a spectroradiometer (USR-45DA; USHIO Inc., Tokyo, Japan).

‘Enrei’ is a well-known edamame cultivar and the second leading soybean cultivar in Japan [38]. As a dwarf tomato cultivar, ‘Micro-Tom’ (*Lycopersicon esculentum*) is widely used to study several aspects of fruit biology in plant science. Therefore, these were selected for the study. The cultivation experiments were conducted in a room with a controlled environment at the Matsudo campus, Chiba University. After germination, seedlings were cultivated under white LED lamps (color temperature of 5000 K; XLX450NHNU LE9; Panasonic Corporation, Osaka, Japan), and the PPFD of the top canopy was set to 200 μmol m^−2^ s^−1^. The spectral photon flux distribution of the white LED lamp was measured using a spectroradiometer (USR-45DA; Ushio Inc., Tokyo, Japan), and the spectral data are shown in Figure 10. In edamame, according to our preliminary experiments, the PPFDs of the top canopy were set to 500 μmol m^−2^ s^−1^ from 12 to 21 DAS using the white lamps. The photoperiods were 16/8 h (light/dark) at the vegetative growth stage and 12/12 h (light/dark) at the reproductive growth stage from 22 DAS. Air temperature, relative humidity, and CO_2_ concentration were set at the same levels as those for dwarf tomatoes, as shown in Table 1. In dwarf tomatoes, cultivation conditions were the same as those reported by Ke et al. [8], and the photoperiod was 16/8 h (light/dark).

At the reproductive growth stage (from 22 to 66 DAS in edamame and from 36 to 82 DAS in dwarf tomatoes), uniform seedlings were transferred to the treatments with different PPFDs. The different PPFD treatments included E300 (E: edamame) and T300 (T: tomato) at a PPFD of 300 μmol m^−2^ s^−1^, E500 and T500 at a PPFD of 500 μmol m^−2^ s^−1^, and E700 and T700 at a PPFD of 700 μmol m^−2^ s^−1^ (Figure S5).

### 4.2. Edible Biomass Space Use Efficacy and Photosynthetic Capacity

EBSUE (g m^−3^) is defined as the crop edible biomass produced per unit of cumulative volume occupied by a plant during a growth period. Therefore, the formula for EBSUE is as follows:

(1) $$EBSUE=\frac{{DW}_{E}}{\sum_{t=0}^{n}V\left(t\right)}=\frac{{DW}_{E}}{{DW}_{n}}\times \frac{{DW}_{n}}{\sum_{t=0}^{n}V\left(t\right)}=F_{E}\times SUE$$

where DW_E_ (g) is the edible dry weight on day n (the harvest day), V(t) (m^3^) is the volume occupied on day t, DW_n_ (g) is the above-ground dry weight on day n, F_E_ (g g^−1^) is the ratio of the dry mass of the edible part to the above-ground dry mass of the plant, and SUE (g m^−3^) is the space use efficacy until day n. V(t) is calculated as follows:

(2) $$V\left(t\right)=S\left(t\right)\times H\left(t\right)$$

where S(t) (m^2^) is the cultivated area of the plant on day t, and h(t) (m) is the plant height on day t.

The dry weights of each organ of above-ground parts in edamame 66 DAS and in tomato 82 DAS were measured. The cultivated area was calculated by a rectangle circumscribing the leaf projection of eight plants. In addition, the cultivated area and height of the plant were measured every 3 days until harvest. The measurements were performed in duplicate.

The dry mass production is strongly correlated with photosynthetic rate and capacity [39,40]. The response of photosynthetic rate to PPFD in edamame 36 DAS and tomato 64 DAS was determined using an LI-6400XT Portable Photosynthesis System (LI-COR Inc., Lincoln, NE, USA) according to the method of Ke et al. [9], and the details are shown in the caption of Figure S3. P_max_ was calculated by fitting light response curves to a nonrectangular hyperbolic function [41]. 

### 4.3. Statistical Analysis

We conducted one-way analysis of variance (ANOVA) using SPSS for Windows (Version 24.0; SPSS Inc., Chicago, IL, USA) to analyze the data. Data presented are the means of four replicates (*n* = 4). To investigate significant differences among treatments, a Tukey–Kramer test was performed at a significance level of *p* < 0.05.

## 5. Conclusions

Our study showed that the EBSUE and SUE increased with increasing PPFD (from 300 to 700 µmol m^−2^ s^−1^) in both the edamame and dwarf tomatoes. The EBSUE increased depending on the increase in the SUE and F_E_ in the edamame and the SUE only in the dwarf tomatoes. Unlike in the edamame, in the dwarf tomatoes, a higher PPFD led to a lower F_E_ due to the limited amount of edible parts (fruit). A higher PPFD resulted in a higher SUE by increasing the dry mass production and decreasing the V in the edamame and by only increasing the dry mass production in the dwarf tomatoes. The fact that the PPFD did not affect the V was attributed to the PPFD hardly affecting plant height of the dwarf tomatoes at the reproductive growth stage. Additionally, the effect of the PPFD on the dry mass production in the edamame was higher than that in the dwarf tomatoes because the higher PPFD more positively affected the P_max_ in the edamame than in the dwarf tomatoes. Furthermore, both the EBSUE and SUE in the dwarf tomatoes were substantially higher than those in the edamame because of the lower plant height and smaller size for the occupied volume. In summary, a high PPFD can improve the EBSUE and SUE of edamame and dwarf tomatoes in different ways at the reproductive growth stage. Moreover, the results of this study provide insights into efficient space and resource utilization in PFALs and vertical farms and may be beneficial in elucidating how PPFD quantitatively affects EBSUE and SUE. However, in determining EBSUE and SUE, the SUE of underground plant parts was not considered. Since nutrients and water are mainly absorbed through roots, underground plant parts may affect dry matter accumulation, SUE, and EBSUE and therefore should be studied in the future.

## Figures and Tables

**Table 1:** Environmental elements during the growth period in edamame and tomato.

Environmental Element	Set Value
Light period (h d^−1^)	12 (edamame) and 16 (tomato)
Air temperature (Light/Dark) (°C)	25/20
Relative humidity (%)	60–70
CO_2_ concentration (μmol mol^−1^)	1000

## Data Availability

Data are contained within the article and Supplementary Materials.

## Supplementary Materials

The following supporting information can be downloaded at https://www.mdpi.com/article/10.3390/plants13131858/s1, Table S1: Effects of photosynthetic photon flux density (PPFD) on numbers of nodes, seeds and pods, and leaf area in edamame 66 d after sowing (DAS); Table S2: Effects of photosynthetic photon flux density (PPFD) on numbers of fruits, leaf area, Brix, and acidity in dwarf tomatoes 82 d after sowing (DAS); Figure S1: Effects of photosynthetic photon flux density (PPFD) on relative space use efficacy (SUE) (A) and dry mass partitioning to edible organs (F_E_) (B) in edamame and dwarf tomatoes; Figure S2: Effects of photosynthetic photon flux density (PPFD) on above-ground dry weight (DW_n_) (A) and accumulated cultivation volume (V) (B) in edamame and dwarf tomatoes; Figure S3: Effects of photosynthetic photon flux density (PPFD) on Pn (A), Ci (C), Cond (E), and Tr (G) in edamame. Effects of photosynthetic photon flux density (PPFD) on Pn (B), Ci (D), Cond (F), and Tr (H) in dwarf tomatoes. Figure S4: Effects of photosynthetic photon flux density (PPFD) on relative edible biomass space use efficacy (EBSUE) (A) and plant height (B) in edamame and tomatoes; Figure S5: Photosynthetic photon flux density (PPFD) treatments in edamame (A) and dwarf tomatoes (B).
