# Supplementary material for: High Photosynthetic Photon Flux Density Differentially Improves Edible Biomass Space Use Efficacy in Edamame and Dwarf Tomato
Source: Plants (Basel). 2024 Jul 5;13(13):1858. doi: 10.3390/plants13131858 (PMC11243976; doi:10.3390/plants13131858)
Supplement: Supplementary file 1 [file plants-13-01858-s001.zip › plants-3010713-supplementary.pdf]

plants

MDPI

Supplementary Materials

High Photosynthetic Photon Flux Density Differentially Improves Edible Biomass Space Use Efficacy in Edamame and Dwarf Tomato

Qingxin Liu [1], Xinglin Ke [1,*], and Eiji Goto [1,2,*]

Table S1. Effects of photosynthetic photon flux density (PPFD) on numbers of nodes, seeds and pods, and leaf area in edamame 66 d after sowing (DAS). Data are shown as the mean ± standard error of the four biological replicates. Lowercase letters within the column indicate significant differences between the treatments as determined using Tukey's HSD test at $p < 0.05$ ($n = 4$).

Treatment	Number of nodes	Number of seeds	Number of pods	Leaf area (cm^2)
E300	13.0 ± 0.6	33 ± 5 b	19 ± 2 b	844.4 ± 150.8 b
E500	13.0 ± 0.4	60 ± 6 b	34 ± 3 b	1469.3 ± 76.3 b
E700	14.0 ± 1.0	113 ± 9 a	62 ± 7 a	2375.8 ± 152.9 a

Table S2. Effects of photosynthetic photon flux density (PPFD) on numbers of fruits, leaf area, Brix, and acidity in dwarf tomatoes 82 d after sowing (DAS). Data are shown as the mean ± standard error of the four biological replicates. Lowercase letters within the column indicate significant differences between the treatments as determined using Tukey's HSD test at $p < 0.05$ ($n = 7$ or 8)

Treatment	Number of fruits	Leaf area (cm^2)	Brix (%)*	Acidity (%)
T300	14.3 ± 0.6 b	340.0 ± 38.1	5.73 ± 0.35	1.29 ± 0.06
T500	15.0 ± 1.4 b	329.0 ± 28.1	5.99 ± 0.21	1.09 ± 0.06
T700	17.4 ± 1.6 a	263.7 ± 43.8	6.61 ± 0.19	0.99 ± 0.08

*Brix and acidity of ripe tomatoes were measured with a pocket Brix-Acidity Meter (PAL-BX|ACID3; Atago Co. Ltd.)

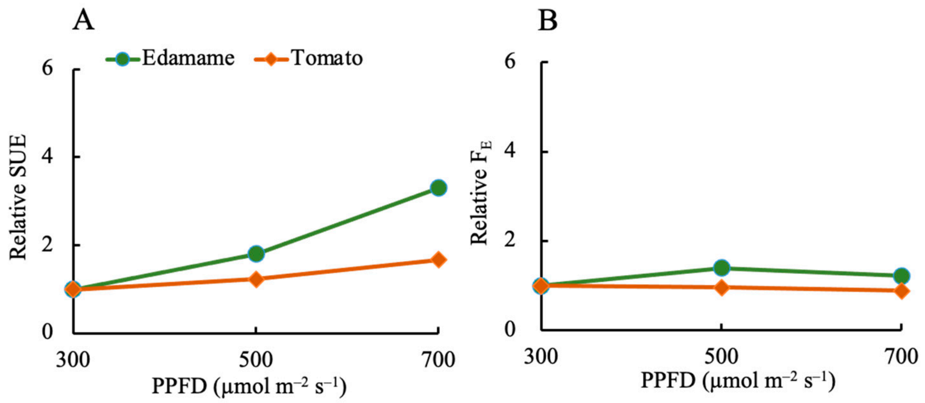

Figure S1. Effects of photosynthetic photon flux density (PPFD) on relative space use efficacy (SUE) (**A**) and dry mass partitioning to edible organs (F_E) (**B**) in edamame and dwarf tomatoes. The data represent multiple increases relative to a PPFD of 300 μmol m^{-2} s^{-1}, with all values at 300 μmol m^{-2} s^{-1} considered 1.

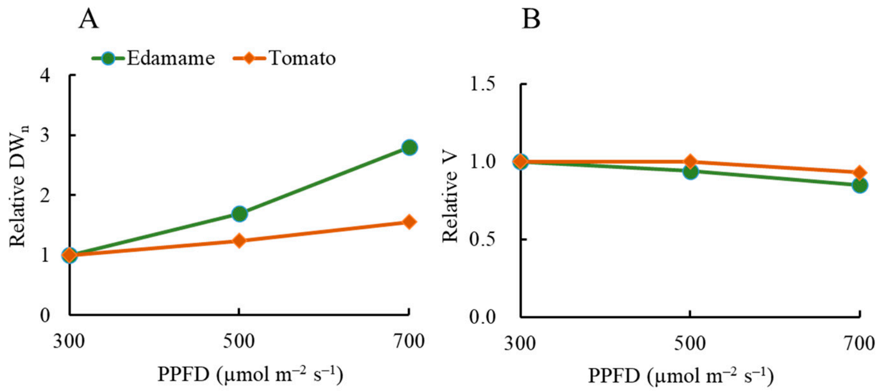

Figure S2. Effects of photosynthetic photon flux density (PPFD) on above-ground dry weight (DW_n) (**A**) and accumulated cultivation volume (V) (**B**) in edamame and dwarf tomatoes. The data represent multiple increases relative to a PPFD of 300 μmol m^{-2} s^{-1}, with all values at 300 μmol m^{-2} s^{-1} considered 1.

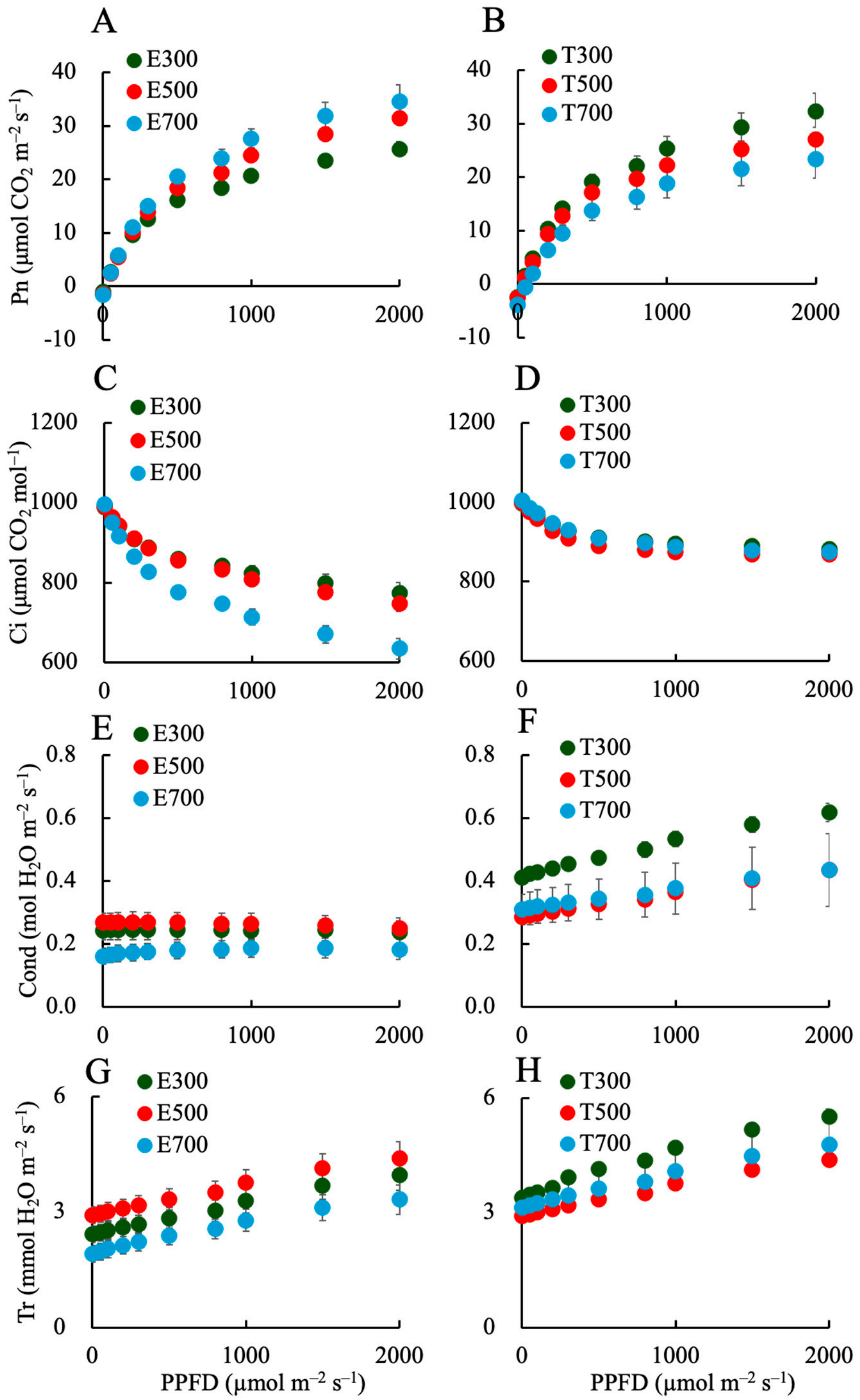

Figure S3. Effects of photosynthetic photon flux density (PPFD) on Pn (**A**), Ci (**C**), Cond (**E**), and Tr (**G**) in edamame. Effects of photosynthetic photon flux density (PPFD) on Pn (**B**), Ci (**D**), Cond (**F**), and Tr (**H**) in dwarf tomatoes. Vertical bars indicate standard error (n = 4). Pn: photosynthetic rate; Ci: intercellular CO_2 concentration; Cond: conductance to H_2O; Tr: transpiration rate.

* Pn, Ci, Cond, and Tr were measured by using a LI-6400XT portable photosynthesis system (LI-COR Inc., Lincoln, NE, USA). The second expanded leaf from the top in edamame at 36 d day after sowing (DAS) and in dwarf tomatoes in 64 DAS were used for the measurement. During the measurements, environmental conditions of the leaf chamber were set as 25 ± 1 °C leaf temperature, 65–70% relative humidity, 1000 μmol mol^{-1} CO_2 concentration, and 500 μmol s^{-1} flow rate of air through the system. The leaves were clamped into the cuvette at 1000 μmol m^{-2} s^{-1} PPFD until the Pn and stomatal conductance were stable. The following PPFD gradient was set at the leaf surface: 2000, 1500, 1000, 800, 500, 300, 200, 100, 50, and 0 μmol m^{-2} s^{-1}.

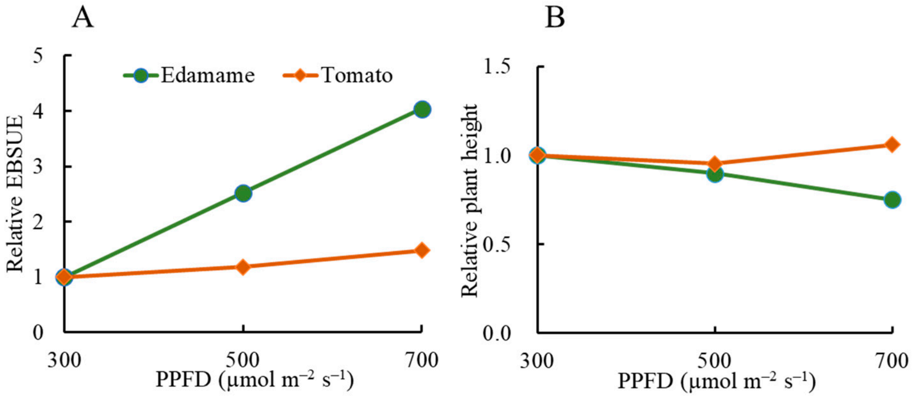

Figure S4. Effects of photosynthetic photon flux density (PPFD) on relative edible biomass space use efficacy (EBSUE) (**A**) and plant height (**B**) in edamame and tomatoes. The data represent multiple increases relative to a PPFD of 300 µmol m^{-2} s^{-1}, with all values at 300 µmol m^{-2} s^{-1} considered 1. The plant height data are for edamame 66 d after sowing (DAS) and dwarf tomatoes 82 DAS.

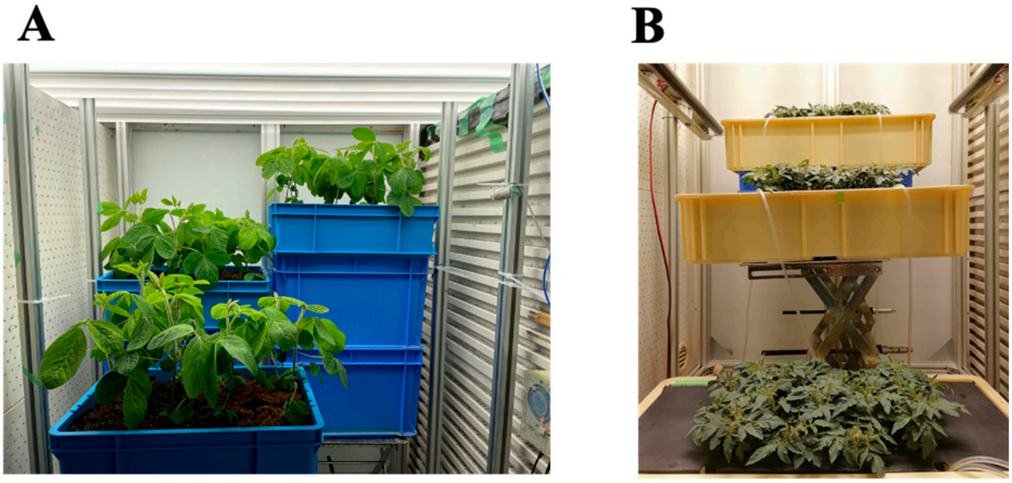

Figure S5. Photosynthetic photon flux density (PPFD) treatments in edamame (**A**) and dwarf tomatoes (**B**).
